# Professional’s Perspectives on Care Management of Young People with Perinatally Acquired HIV during Transition: A Qualitative Study in Adult Care Setting

**DOI:** 10.1371/journal.pone.0169782

**Published:** 2017-01-23

**Authors:** Enora Le Roux, Serge Gottot, Camille Aupiais, Thomas Girard, Maria Teixeira, Corinne Alberti

**Affiliations:** 1 Université Paris Diderot, Sorbonne Paris Cité, ECEVE, UMRS 1123, Paris, France; 2 Inserm, ECEVE U1123 and CIC-EC, CIC 1426, Paris, France; 3 Assistance Publique-Hôpitaux de Paris, Hôpital Robert Debré, Unité d’épidémiologie clinique, Paris, France; 4 Assistance Publique-Hôpitaux de Paris, Hôpital de l'Hôtel-Dieu, Unité Guy Mocquet, Paris, France; Centre Hospitalier Universitaire Vaudois, FRANCE

## Abstract

**Background:**

Increasing numbers of young people with perinatally acquired HIV are surviving to adulthood. When they come of age, they leave pediatric services in which they were followed and have to be transferred to the adult health care system. Difficulties in adaptation to adult care and the numbers of young people lost to follow up after transfer to adult care have been reported. This transition phase and their retention in adult care are crucial in maintaining the clinical status of these young with HIV in adulthood. Our study aimed to explore how HIV professionals working in adult care perceive and adapt their practices to young people in transition.

**Methods:**

Qualitative interviews were conducted with 18 health and social services professionals in hospitals or patient associations in France. A thematic analysis was conducted.

**Results:**

Adult care professionals were found to be making a distinction between these young people and their patients who were infected during adulthood. On the basis of the healthcare teams’ experience, a simplified categorization of these young people into four levels can be used: those “who have everything good”; those who have some deficiencies that must be addressed; those “who have everything bad”; and those lost to follow up. Professionals interviewed highlighted the difficulties they encountered with young people in transition. Three types of problematic situations were identified: problems of acceptance of the disease; communication problems; and problems of disorientation in the new care environment.

**Conclusions:**

Despite the lack of specific training or national policy recommendations for the integration of young people with perinatally acquired HIV into adult services, all the adult healthcare teams interviewed tried to adapt their practice to this population. The results suggested that professional involvement during transition should depend on the characteristics of the patient, not be limited to a single transition model and that a dedicated structure for transition care is not appropriate for all young people.

## Introduction

Early infant diagnosis and therapeutic innovations mean that individuals with perinatally acquired human immunodeficiency virus (PHIV) or HIV acquired during early childhood are increasingly surviving into adolescence and adulthood [1].

The young people affected are still very small in numbers in northern countries. Given the existing databases, we can only estimate the numbers of young people with HIV acquired during the perinatal period or in early childhood. For example, in 2014 in France, 3930 children and adolescents (up to 14 years) benefit from exemption from their care costs for infection with HIV thanks to universal health insurance, they represent less than 4% of HIV population [2]. In the United States 3121 adolescents (up to 14 years) were living with diagnosed HIV infection in 2013—less than 1% of the diagnosed HIV population [3]. During adolescence, these young people receiving care from childhood must continue their care pathway through transitioning from pediatric to adult services.

This health care transition is defined as the “purposeful, planned movement of adolescents and young adults with chronic physical and medical conditions from child-centered to adult-oriented health care systems.” [4]. This phase is recognized as a high-risk period for the health status of young people who can experience difficulty making the transition to the adult health system [5]. The reasons of poor adaptation in adult care are multiple and may be common to a set of young people with chronic diseases: in adult care the professional caregivers may change at each visit, consultations are more focused on clinical outcomes rather than psychosocial or developmental concerns, there is less warmth communication between patients and professionals, there is a need for the young to be autonomous in its illness management which is not necessarily the case in pediatric services [6]. This can lead to disruption in medical follow-up. The literature revealed some significant adverse health consequences related to lack of continuity of care or resort to emergency services following a bad transition [7].

In different chronic conditions, many studies have highlighted these consequences of transition, such as the increase of organ rejection in kidney transplant adolescents [8], the rise of HbA1C in young people with type 1 diabetes mellitus (T1DM) [9], or the increase of disease activity in chronic rheumatic disease [10].

Among young people with PHIV, transition is a crucial step for the achievement of an optimal health status in adulthood too. It requires therapeutic and care continuity between pediatric and adult services [11]. A study in England has identified an increased crude mortality rate following transfer to adult care of young people with PHIV [12]. Such findings highlight the need to ensure the healthcare transition success. However, difficulties encountered in delivering appropriate health care for young people with HIV have been widely identified. The United Nations Program on HIV/AIDS (UNAIDS) reports that there is poor prioritization of adolescents in national plans for scaling-up HIV testing and treatment services [13]. The World Health Organization (WHO) has also found that health workers, even those experienced in caring for adults with HIV, are often ill-equipped to support the healthcare needs of adolescents [14].

The confrontation of HIV-specialist professional working in adult care with youth is unusual, this population represents generally less than 4% of its active list of patients which is not the case for a set of other chronic diseases where a larger proportion of patients followed in adult care have developed their illness since childhood. Besides the rarity of the situation, this population is characterized by its unique issues that is necessary to take into account [15]. Young people with HIV addresses specific challenges particularly because of its long history with the infection, taboo and secret surrounding the disease since his birth, the parent’s guilt or even its socioeconomic status often linked with family migration stories.

The findings of a care system currently unsuitable for young is alarming since poorly adapted systems are recognized as barriers to optimal engagement in the care of patients with HIV [16]. Conversely, communication between adolescent and adult providers, and age- and developmentally-appropriate care, are considered key facilitators of successful healthcare transition [17].

While the WHO has developed a toolkit for transition of care for adolescents living with HIV [18] and the US has proposed guidelines [19] some countries such as France do not have national recommendations for healthcare transition of young people with PHIV.

Our research aimed to explore how healthcare teams with experience in caring for adults with HIV perceive young people with PHIV in transition and adapt their practices to ensure its success.

## Materials and Methods

### Participants

Participants were health and social care professionals with experience of transition in HIV care, defined as the involvement in at least 5 cases of young people with PHIV in transition during their career. In France, HIV-positive patients are almost exclusively follow-up in hospital sector and so this is the setting in which was mainly conducted our recruitment. Initial recruitment was carried out in a pediatric research hospital serving patients of diverse ethno-cultural origins, from central and suburban Paris. The hospital is situated in the region of metropolitan France with the highest HIV prevalence rate [20, 21]. The head of the pediatrics department authorized the identification of the first participants who were professionals from other hospitals and associations with which he collaborated or who were known to be particularly involved in the care of young people with PHIV. Participants identified in this primary listing were used as informants in a snowball procedure. They were asked if they had contacts with professionals involved in healthcare transition of young people with PHIV and if they were willing to provide us directly with potential respondents’ contact details. All these recommended professionals were invited to participate, and recruitment of participants continued based on diversification until empirical saturation of the data appeared to have been reached. Saturation means that no additional data are being found whereby the researcher can develop the properties of the category. Seeing similar instances over and over again, the researcher becomes empirically confident that a category is saturated [22]. Our sample was constructed with the aim of providing a comprehensive overview of situations and problems encountered by professionals involved in transition. Thus, in addition to hospitals and teaching hospitals from Paris and suburbs, one youth health unit and one association for persons living with HIV/AIDS “Dessine moi un mouton” participated. The youth health unit is attached to a hospital for adults and is dedicated to the medical care of adolescents and young adults experiencing familial, social or educational breakdown, whether HIV-infected or not. The association offers young people affected by HIV/AIDS a place where they can come together and participate in activities planned by a team of animators, social and educational assistants and psychologists. Between February 2014 and February 2015 we contacted 20 professionals and 18 were interviewed: 1 nurse supervisor did not respond, and 1 patient association responded that their population did not match our target of young people with PHIV. (See Table 1 for characteristics of participants)

**Table 1:** Participants characteristics (n = 18).

	**Numbers**	**(%)**
**Gender**		
Female	11	(61)
Male	7	(39)
**Education**		
Medical doctor	9	(50)
Nurse	3	(17)
Auxiliary nurse	1	(<6)
Psychologist	4	(22)
Social worker	1	(<6)
**Workplace**		
Adult healthcare services	8	(44)
Youth health unit	8	(44)
Patients association	2	(11)
	**Median**	**(range)**
**Years of experience with HIV**		
Medical professionals	21	(4–32)
Paramedical and social professionals	8	(1–22)

### Procedure and interview protocol

Semi-structured face to face interviews with professionals were conducted by either one or two of the authors (EL, SG), who were trained in interviewing techniques. Researchers who conducted the interviews and the coding process are graduated in public health. Interviewers were one PhD candidate and one senior lecturer, affiliated to a research team working in particular on issues of children and adolescents with chronic diseases. Interviews were mostly individual (n = 14/16, 87%), and took place at the participants’ workplaces, enabling the researchers to observe the environments in which young people experience transition. Two interviews were not individual and were conducted with two interviewees: these were with the professional staff of the patient association and with nurses of the youth health unit. This choice was made by the professionals interviewed, who usually work together. An interview guide was developed by reviewing the transition-related literature, and was adapted after first interviews with professionals to collect the different perspectives of professionals regarding their experience with young people during healthcare transition, the elements required to ensure a good transition for these patients, the problems they had encountered and the means implemented to try to correct them. Interviews were audio-recorded or documented in notes taken by the interviewer and digitalized. Interviews lasted 25–70 minutes, depending on the time each participant could offer and the experiences he/she had to relate.

### Regulatory and ethical aspects

As part of a non-interventional study and not including patients, written consent is not required according to the French Public Health Code (Articles L.1121-1 and R 1121–2). However, information has been delivered according to the Article L1122-1. An information brief on the study was provided to all participants. This procedure was described in the protocol submitted to the ethics committee and approved by the local ethics committee of the university hospital Robert Debré (Comité consultatif d’éthique local de hôpital universitaire Robert-Debré, committee's advice number 2014.152). Required permission from relevant authorities have been obtained (Advisory Committee for Data Processing in Health Research (CCTIRS) and French Data Protection Authority (CNIL)). Oral informed consents were obtained from all participants for the use of data for research and for recording interviews. Information was delivered since the initial contact and renewed at the time of starting the interview where we asked the participants to affirm their consent to participate in this research and to be registered during the interview. The ethics committee approved this consent procedure.

### Coding and analysis

Interviews were transcribed verbatim by a professional transcriber and digitalized notes taken during the interviews were coded. Contents were analyzed with NVivo® 10 software. Codes were generated through a process of inductive identification of themes [23]. Transcripts were first coded independently by two of the authors (EL, SG) who had formal training in the analysis of qualitative data. Emerging themes and analytical framework were discussed by the researchers after each working session to reach a consensus on coding. An agreed preliminary coding framework was presented to a multidisciplinary group from the “Société Française de Lutte contre le SIDA” in a workshop on the theme “HIV-positive teenagers” with teams from France, French overseas departments, and Francophone countries who had experience of transition. Analyses were refined following feedback at this workshop.

To maintain anonymity all names have been changed.

For more methods details, see the COREQ Checklist [24] in S1 Checklist.

### Identification of themes

After coding and categorizing data an analytical framework was formed. It included 4 main groups of themes: those related to the description of the patients by the interviewees, those related to the problems encountered during the transition, those related to the elements acting for a smooth transition and those related to the impact of transition. Only a portion of the identified themes that are those related to professional’s perspectives on care management of young people with perinatally acquired HIV during transition are presented in the results section. The themes identified are derived either from topics proposed by the interviewed professionals that researchers inform with the content of others interviews, or themes that had newly emerged from the content of the interviews. Themes appear in quotation marks in the text of the results’ section.

## Results

We identified themes related to professional’s perspectives on care management of young people in transition. The themes presented concern the categorization of patients in transition made by health professionals, the practices proposed to deal with the transition problems and the difficulties encountered by professionals who are facing youths in transition.

### Characterisation of patients transitioning from pediatrics to adult services by professionals

All the adult care professionals have expressed the “difference in feeling or care experience” they had with young adult patients who started their care in pediatric services, and those who started their care in adult services.

*“But the mother-child transmission ones are my favorites”* (Nicole, medical doctor, adult care)

[About PHIV young people] *“But it’s true that it’s harder for me*, *they are still always more difficult”* (Christian, medical doctor, adult care)

Another theme that emerged was “the difficult patient”. According to interviewed caregivers, youth infected since childhood are globally more difficult than other patients infected and medically treated since adulthood.

*«It’s difficult because they are kids who frequently are fed up*, *fed up of medications*, *they don’t want to take their medicine »* (Nicole, doctor, adult services)

*“This is the limit which is difficult sometimes not like with adults who understand that sometimes we are very limited regarding the treatment choice and that at least for some time they have to make some efforts*. *He [a teenager] wasn’t capable to make these efforts*.*”* (Christian, doctor, adult services)

The experiences of adult care providers suggest that young adults in transition from pediatric care can be grouped into categories. The four labels suggested by one of the interviewees were informed with the content of other interviews to create themes.

The theme “good cases” described the first category of young people which are characterised by school education, family around them, well-adapted pediatric therapeutic choices, few antiretroviral medicines to takein adulthood, good adherence, a degree of autonomy, and some life projects.

*“There is a young boy that doesn’t have parents but he has met good people throughout his childhood*. *When his parents were sick he went to foster care but he remained in contact with his parents… He was in a foster home that didn’t know anything about HIV but they adapted*. *That is his family at heart*. *Since then he works*, *he is autonomous*, *he fell in love two years ago*, *and they plan to have a baby”* (Catherine, nurse practitioner, adolescent and young adult care)

According to the professionals’ experience, support provided by a specific structure dedicated to care for adolescents and young adults during transition is not necessary for these patients

*“This was a young man who saw* [the adult doctor] *once here* [in the youth health unit] *and found it was a stage which didn’t really concern him*, *so he went straight into the adult system” (*Catherine, nurse practitioner, adolescent and young adult care)

The second theme included young people considered as “intermediate cases”, whose characteristics are not bad but not perfect, with current follow-up but also some weaknesses such as therapeutic compliance, consultation attendance or global health knowledge. An available and socially supportive team was suggested as a means to nurture young people’s capacity to invest in their care, and to recognize the right time to work with them.

*“Then there are all the intermediate cases*, *those who are not so bad but not perfect*, *who have a pretty good medication compliance but still have big weaknesses*, *who need to be somewhat stimulated”* (Richard, medical doctor, adults care)

The theme “poor cases” described the third category of patients characterized by little or no education, no family support, non-compliance with treatment, no comprehension of health issues related to their health status, inability to attend planned consultations.

[About young people of this category] *“I am categorizing*, *not stigmatizing anyone*, *but generally what I say is [they have] ‘a 300 word vocabulary*, *a CD4 count of 300 and a viral load of 30*,*000 copies [that indicate poor clinical outcomes]”* (Richard, medical doctor, adult care)

*“A young French boy with parents from Algeria who were drug addicts*. *The mother died when he was five or six years old*, *and his father took care of him*, *he went with him to his doctor’s appointments*. *His father died when he was 12 or 13 years old*, *and then he was raised by his grandparents who were not very loving*. *It was a bit an outsider of the family*, *he had an older sister that was not infected*, *she try to took care of him sometime but she had too much to do elsewhere*. *He was totally non-adherent*. *For many years he had CD4 counts very low*, *really like CD4 of 0 [indicating a very poor clinical results]*, *but he still had a conserved health state*. *He had some oesophageal candidiasis that resolved quickly in two or three days of hospitalization We tried to start a treatment*, *and he stopped once he left the hospital*. *I saw him again 3 or 6 months later […] Now he was in prison*, *and it has been a year and a half or two years without any news”* (Christian, doctor, adult services)

For these young adults, according to the healthcare teams, the only rule is to be as available as possible, offering a structure that provides flexibility, an element of self-service, but which also educates them in the imperatives of a health structure and of health awareness.

The fourth and final category was grouped under the theme “extreme cases” which describes the young with which it is extremely difficult to establish contact, and who usually end up being lost to follow-up.

*“Those who we are unable to see*, *despite all the appointment booking*, *all the telephone reminders*, *we're a little desperate”* (Richard, medical doctor, adult care)

### Means to handle healthcare transition and arrival in HIV adult services

Among the elements proposed by the interviewees to allow smoother transition from pediatrics into adult services we have identified two main themes: "strategies for the social support of the patients since childhood" and "strategies to ensure continuity of care between pediatric and adult services” that appeared as elementary key points to implement in pediatrics according to professionals from adult services (table 2).

**Table 2:** Transition key points identified in the interviews.

Actions proposed	Professionals involved
***Themes*: *Strategies for the social support of the patients since childhood***	
● Provide necessary accompaniment and counseling to parents/tutors that would enable them to support their child all along his journey with illness and care	● Pediatrics and adult services teams
● Identified soon enough the person (parents, siblings, aunt…) who should be the link between healthcare providers and the young if the latter disengaged of his care and boost young to going back to it	● Pediatrics teams
***Themes*: *Strategies to ensure continuity of care between pediatric and adult services***	
● Integrate transition targets all along care pathway	● Pediatrics teams
● Discriminate youths who have special needs at the time of transition (ex: special structure)	● Pediatrics teams and adult services teams for repechage
● Prepare with the young a transition report aimed at adult teams with:	● Pediatrics teams
- Biomedical data and treatment histories	
- Important elements about backgrounds and life environment	
- The contact information of the person identified as the link between healthcare providers and the young	
● Identify a network allowing effective relays between pediatrics and adult services who take over HIV AYA patients	● Health policy makers, providers and health organization managers

Relatively to “practices applied in adult services”, things were more heterogeneous. In adult services there was no official program implemented for these young people. Professionals reported unofficially implemented practices that adapt to the specific needs using available resources and knowledge.

*“He could have a treatment once a day*, *three pills*, *but it’s hell*. *It’s hell even if we try to do an arrangement one time*, *he came in the service everyday take his pills*. *We prepared food*, *there was the nurse*, *the psychologist*, *me and all the patient education team*, *we stayed with him when he was eating we put mint syrup in his water for him to swallow his pills*, *he took them in front of us*. *He did this for 15 days*, *and then he disappeared” (*Nicole, doctor, adult service)

*“We did a group with some adolescents here with 2 psychologists*, *there were 2 because there was one from pediatrics and one from my service and we have formed a group from nothing*, *that tried to bring together adolescents”* (William, doctor, adult services)

*“We proposed recreational things*, *we want to propose—but we will see if it is financed—to do a little weekend where we rent accommodation*, *we take ten teenagers from our service… We have some ideas about the activities for the weekend*, *we have done some dinners with 5–6 teenagers in a pizzeria and we talked about this idea… This helped to establish a link”* (Victor, doctor, adult service)

### Relationships between adult providers and young people in transition

Three themes categories have emerged from discussion on major problems faced by health professionals with young people in transition. They are: difficulties of young people in relation to illness, difficulty in linking young people with their new adult care providers and culture clash between the new environment and the young people.

#### Difficulties of young people in relation to illness

The themes of the first category described young people denying their disease or disinvesting in their care at the time of transition. They often miss their consultations, and refuse or forget to take medication. This may be part of “adolescent behavior” to question what he always did without asking any question during his childhood:

[Describing, according to his view, the thoughts of these patients about medication] *“I was told it was good*, *that it worked*, *and now I want to see if it’s true”* (David, psychologist, adolescent and young adult care)

It may also reflect “non-appropriation of the disease”:

*“His first thought was ‘I don’t want to go there* [to the infectious diseases] *because everyone will know what I’ve got*, *everyone has what I have”* (Catherine, adolescent and young adult care, nurse practitioner

*“After finishing with the pediatrician*, *she had difficulties with the adult infectious disease specialist but not solely because of relationship problems, I also feel that it was a way to forget the HIV label”* (Catherine, adolescent and young adult care, nurse practitioner)

For these patients, solutions have been developed by teams to anticipate the problem. Some support and work with parents on issues of secrecy and shame which may be passed on to PHIV children and on the representation and place in daily family life of the disease and its treatment.

[speaking of what she recommends to parents] *“Take medication in front of them*, *if they ask questions*: *answer [*…*]talk about it*, *so that the child incorporates the fact that taking treatment for HIV is something natural”*. (Nicole, medical doctor, adult care)

Others provide space and opportunities for parents and young people to verbalize their concerns, individually or with peers in clinical services or via an association.

Others physically offer them an intermediate care location which is not specific to the HIV population but dedicated to adolescent and young adult patients. This enables them to move away from pediatrics, and gradually initiates their transfer to adult care by providing a transitional place.

#### Difficulty in linking young people with their new adult care providers

The themes of the second category concerns difficulty in linking young people with their new adult care providers. This may result from a “lack of comprehension of adolescent attitudes”:

*“I even wondered if she might be neurologically a bit weak*, *you know*. *Because… but in fact she was exactly the type you get with teenagers*, *they seem stupid but really it’s that…*. *well*, *it’s just being a teenager*, *the typical teenager*, *who won’t say anything*, *who answers rudely”* (Christian, medical doctor, adult care)

*“How to talk to an adult who seems like a child*, *who has some sides to them that are still very like a child”* (Melanie, psychologist, adult care)

This may also result from an “inability to establish suitable communication”:

*“When they become adults*, *they have multi-resistant viruses*. *They are completely… they are against everybody in general*, *they stop their treatment*, *they restart*, *they stop*, *and they restart*. *Consequently… their situations are very very difficult*, *on a medical level and very difficult on a relational level because we have a lot of difficulties to*…*We don’t succeed*.*”* (Diane, medical doctor, adults care)

*“Last year there was a young boy that died because he didn’t take his medicine*, *he died even though he had a virus without any particular mutation or resistance*, *but he died because he didn’t take his treatment*. *Even though he was in a medical structure 3 times per week for his dialysis treatment*, *and it was a terrible failure for us because I am convinced that the only problem was the inability to communicate between him and I and between professional caregivers in general*.*”* (Victor, doctor, adult services)

The situation of “professionals inadequately equipped to address the complex psychosocial needs to these patients” also emerged as a theme.

*“We are desperate to see kids that come like Celia*, *pregnant at 18 years […] she took her treatment during her pregnancy but she faced difficulties*, *moreover her virus was multi-resistant to treatment […] The baby was born the week of her 18*^*th*^ *birthday*, *and having the baby didn’t motivate her to begin taking her treatment*, *she didn’t tell herself ‘I have to raise my child alone*, *and I need to take my treatment’*. *It wasn’t reason enough*. *[…] They are in such an emotional and economic slump and everything else*. *And this is the same case for her mother*, *she wasn’t present with her she was also non-adherent… her father that wasn’ t present*, *they don’t talk their reality*, *they act as if it doesn’t exist*…*She doesn’t want to see a psychologist*…*and that’s it…”* (Nicole, medical doctor, adults care)

The difficulties in care management can be partly explained by the “lack of training in the initial course of adult doctors” for the taking over of teenagers

“They are still more difficult… I had even participated in a “workshop” on teenagers: “HIV and teenagers” 2 years ago. It was more an exchange of experiences than a training because there was not any particular formation” (Christian, medical doctor, adults care)

*“The first young girl that I received about 15 years ago*, *I didn’t feel like I had the training to manage her care*. *So I did a DIU [a training available to professionals who wish to train for new specialties]*. *I did the one which is for adolescent care that was in Paris for two years*. *It was interesting because there was a lot of … it was the psychological training that I was missing”* (William, medical doctor, adults care)

Professional opinion is that such situations lead to medical and relational difficulties with a high risk of dropping out. Practitioners proposed solutions such as transferring these young people to experienced teams, or to train those who encounter difficulties. Some adult teams advocated a gradual approach, taking time to discuss non-medical aspects and to identify the right moment for the young person to take ownership of his or her HIV status, before focusing on technical issues.

*“Those lost to follow up*, *it’s because I think the first consultations were not reassuring for the patient*, *not enough explanation*, *maybe going too fast in talking about treatment*, *a very technical way of speaking [*…*] the first consultations are fundamental for the future*, *and when it is going well*, *when we don’t rush them by imposing things*, *frankly I think it works well”* (Victor, medical doctor, adult care)

Professionals develop some practices to “establish trusting relationships” with these young patients.

For example, the doctors accept to compromise with recommendations on the occasion of the first consultations or when young people need a break

*“I think it is really that*, *to give them confidence*, *after individual consultations with the patients*. *The first sessions are fundamental for the future*, *and when it goes well*, *when we don’t push them to do things*, *that works the best*, *and then not being upset with young patients when they miss their consultation or because they didn’t do their tests or take their medication*, *that’s it*.*”* (Victor, medical doctor, adult care)

*“We have done years like that between 18 and 23/24 years old*, *where she decided not to take her medication*, *and then she told it me “Ok*, *I stopped everything because I was fed up before Christmas*, *and then I started again 2 days ago” It was ok because she didn’t develop mutations because she stopped everything at once*. *This is not recommended at all but in all cases we have to adapt to them*, *it is so complicated*.*”* (Nicole, medical doctor, adult care)

With the same idea of overcoming difficulty in establish relationships to young people, some care providers organize meetings outside consultations.

*“3 or 4 times a year we have thematic meetings*, *which are open–there are leaflets in the consultation rooms and also in the pharmacy—[…] there is a theme to attract people*, *and there are at least 2 young people who came to these meetings [*…*] for them I am convinced it was somewhat the opening of the chrysalis”* (Richard, medical doctor, adults care)

Others share their direct line phone number or identify a person in the new adult care team to create a relationship with the young patient

*“It may often be someone like a duty nurse who creates a relationship*, *not because they don’t create a relationship with us*, *but because we are less available*, *she* [the nurse] *says "anyway I'll call you in two days*, *or you call me”" she has a direct phone number so it's a little trick*.*”* (Nicole, medical doctor, adult care)

#### Culture clash between the new environment and the young people

The themes of the third category concern the problematic situation where “the new environment and the young people do not match”; there is a culture clash. Interviewees reported that first contact with the adult service, where medical and technical language prevails, where care workers are less patient, available or attentive, where young patients are considered as independent adults, and where the population of the waiting room is very different, can be a rude shock.

*“You get this very pediatric discourse […*.*]*, *how there is a little virus which is a problem*, *which we put to sleep*, *which has to be watched*. *But with this kind of brutal transition into adolescence we see that suddenly*, *[…] you are dealing with something else*, *with an approach which suddenly becomes more medical and*, *in the end*, *much more anxiety-provoking*” (David, psychologist, adolescent and young adult care)

*“A young man of 20 who was accosted in the waiting room by a transvestite*…*So it happens*…*we have a colleague who told us “*[the patient] *came to hide in my office because he* [a transvestite] *picked him up”*. *So all of that is what I'm referring to when I say “violence”*. *I don’t know if they are prepared for that*. *Regardless of how they are prepared before*, *I think they're not prepared for that”* (Melanie, psychologist, adult care)

To reduce the gap between these two worlds, some pediatric and adult teams take part in joint reflection groups, consultations or training on the specificities of care for young adults. Transition files to share data about patients’ knowledge and relationship to illness, amongst other aspects, have also been proposed.

## Discussion

Young people with PHIV constitute the entirety of the active caseload of pediatric healthcare teams working with HIV patients, whereas in adult health care teams they represent a very small proportion of the patients. We have found that professionals working in adults care completely distinguish these young people from their patients infected since adulthood, as it has been recommended [19]. While most professionals pay particular attention to these patients, all of them recognize that they are globally difficult. This is consistent with the description of these patients as a unique patient sub-populations made from a global review about perinatally HIV-infected adolescents. There is mention of the particularly complex adherence in adolescence, socio-economic pressures related to orphanhood, neurocognitive deficits associated with chronic and severe HIV infection, stigma, discrimination and socio-economic levels of families [25].”Difficult patient” is here defined as one who makes healthcare teams feel ineffective [26], because of non-compliance with treatment or resistance to forming an effective alliance with their medical provider, as reported in pain practice [27]. We can also discern here the concept of “bad patients” [28], as patients who fail to validate clinicians' sense of themselves as effective professionals, who threaten their control, and/or who create fruitless work [26]. Patients can be evaluated as “bad” according to their diseases, their behavior, their social backgrounds, their attitudes and/or staff attitudes [28]. Some adolescents may be considered as bad patients by healthcare teams who are unfamiliar with this age group, particularly in the field of HIV where non-adherence to medication is commonly reported [29].

Difficulties in forming an effective alliance in care also suggest the idea of the “difficult doctor”, one who may be highly frustrated because of being inadequately equipped to address the complex psychosocial needs of his or her patients [30]. In the context of transition, poor evaluation of adolescents’ abilities to receive and process information and lack of communication are reported as the greatest barriers to successful transition to HIV adult care [14]. Moreover, it has been shown that patient-clinician relationships are predictive of adherence in HIV patients [31] so this needs to be considered during transition. In our study, adult HIV care workers recognized that the relationship with these young patients requires careful and sensitive negotiation to ensure that trust is established, as reported in more detail elsewhere [32]. To better enhance relationships between patients in transition and their professional caregivers improvements in the following areas could be taken into account: ensure that the transition to adult care is made to professionals with a particular sensitivity to the care of adolescents and young adults; reinforce mutual learning between pediatric and adult care professionals to transfer knowledge for adolescent care which is between their two areas of expertise (for instance in carrying joint consultations); identify in each service one professional as a resource person—sensitive to adolescents and young people—who can act as an intermediary between the new professional caregiver and the patient; when young people who have to leave pediatrics are not sufficiently ready to move directly into the adult sector propose a longer period of transition via an intermediary adolescent specialist.

Our research took place in the French context, where there are no national guidelines for a structured healthcare transition for young people with PHIV. The transition process—the existence of a pediatric preparation, its contents, the age at transfer—is highly dependent on healthcare settings. Because of the French welfare model, insurance or access to medication do not appear as major sources of failure, as is the case in other countries with different systems [33]. The solutions implemented by the teams were developed in this context but may feed into the reflection in other countries. The patient categories we report here are broad and schematic but bring together in 4 groups the majority of patients. Individual young patients do not match every feature of a category but in overall terms correspond to one of them. The categories have not been proposed as a hierarchical ranking but as ways to better guide adaptation of young patient handover during the transition.

In our context of young people with HIV in transition to adult care in France, doctors were the professionals who were mostly concerned, nurses were involved but in a lesser extent, and other paramedical or social professionals were rarely involved with the youth in transition. Doctors were mostly those who allowed to distinguish the 4 categories and give the first elements of description. Data provided by the nurses allowed us to enrich and illustrate the different profiles. Finally, because psychologists and social workers do not interact with all patients—they usually do not see those who are "all good" and those who are “extreme / dropouts”—their perspective was different and more limited but helped nourish some of the categories. One limitation of this work is that the perspectives of other people involved in care transitions such as patients, caregivers, or professional providers from other sectors were not explored in this specific study. These aspects could enrich the understanding of care management of young people with PHIV and could be important to keep in mind when thinking about youth in transition.

There are a number of prospects for this work, for example by studying the different way in which patient may be “classified” according to various profiles of professionals or setting (for instance in pediatrics or adult sector). Indeed, it is likely that professionals with paramedical, medical or social backgrounds may have different criteria to understand the situation and categorize patients. These criteria may be clinical, social, emotional, behavioral. In our study categories are based on a set of these criteria.

A possible direction for future research might be to analyze the effectiveness of personalized transition care for young people with PHIV which uses the proposed categories to provide differential and adapted support for each young person.

These studies could provide an assessment of the effectiveness of interventions and elements for standardisation of "differential transitional care". Based on the evidence generated by these evaluation studies and existing recommendations, standardisation of transition practices could be undertaken so that young people in transition benefit from equitable and consistent transition care based on demonstrated effectiveness rather than health centers' individual decisions. Another prospect could be based on the finding that caregivers had very different ways to address problems with patients during care transition and way of perceiving these patients. It has been reported elsewhere (26), that moral appraisals may vary according to patient characteristics, clinician characteristics, tasks, organisational factors, and play a role in the quality of relationships with patients. The concept of emotional intelligence [34], seems particularly relevant to study. It represents the ability to monitor one’s emotions as well as others’, to discriminate among them, and to use this information to guide one’s thinking and actions [35]. These competences appear to correlate positively with patient care and communication [34]. Our findings, and the association of good patient-provider relationships with positive health behaviors in HIV [36], suggest the importance of addressing emotional intelligence in HIV providers. A last prospect to this work would be to study the extent of these classifications to other groups of patients.

## Conclusion

Our research has explored how adult healthcare teams adapt their practices to young people with PHIV during transition. We found a lack of formal training in adolescent issues, but also discovered adapted practices being unofficially implemented using available resources and knowledge. Most of these practices are not routinely offered but are implemented according to the profile of the young patient or to the origins of the transition problem: relational, acceptance of illness, failure to navigate services. Our findings show the importance for professionals to adjust their practices to be consistent with the overall needs of adolescents. It is also important to moderate the support during transition for different profiles of young people and construct a suitable and satisfactory care relationship–that is an essential element to transition success.

Results assert that there is a need to provide differential transition care to provide the best support to all young people. The choice of the type of care best suited to the particular needs of the patients may be facilitate based on a categorization of patients in groups depending on their characteristics. This proposal is based on data obtained from adult care professionals. However, it should be confirmed with the views of all of the other actors involved in transition.

Evaluation of needs and discussion about the different transition plans have to be done with professional’s care team, parents / guardians and of course the teenager himself. There is a need to pursue the reflection, on how to offer flexible transition programs taking into account the diversity of PHIV patients profiles. Indeed, as well as a "light" program may not be sufficient for patients who most need support, an extensive and intensive program may not be suitable for young people that are already being autonomous and ready to adult care. For reasons of feasibility and cost, but also to ensure efficient and satisfactory transition for patients it is important to deliver adapted transition care that have to be differential according to characteristics and needs of patients. Likewise, it is important to provide standardised and common practices during transition for patients who have the same needs, to access well adapted care and increase patient equity. The assessment of differentiated approaches on efficiency criteria and cost-effectiveness could be undertaken.

## Supporting Information

S1 Checklist Consolidated criteria for reporting qualitative studies (COREQ)–Checklist. (DOCX)

## Abbreviations

AIDS: Acquired Immune Deficiency Syndrome
HIV: Human Immunodeficiency Virus
PHIV: Perinatally Acquired Human Immunodeficiency Virus
UNAIDS: United Nations Program on HIV/AIDS
WHO: World Health Organization

## References

[1] Hazra R, Siberry GK, Mofenson LM. Growing up with HIV: children, adolescents, and young adults with perinatally acquired HIV infection. Annu Rev Med. 2010;61:169–85. 10.1146/annurev.med.050108.151127 19622036

[2] Long-term conditions prevalence—Year 2014—French insurance “ameli” [Internet]. Available from: http://www.ameli.fr/l-assurance-maladie/statistiques-et-publications/donnees-statistiques/affection-de-longue-duree-ald/prevalence/prevalence-des-ald-en-2014.php

[3] Centers for Disease Control and Prevention. HIV Surveillance Report, 2014; vol. 26. Diagnoses of HIV Infection in the United States and Dependent Areas [Internet]. Available from: http://www.cdc.gov/hiv/pdf/library/reports/surveillance/cdc-hiv-surveillance-report-us.pdf

[4] Blum RW. Introduction. Improving transition for adolescents with special health care needs from pediatric to adult-centered health care. Pediatrics. 2002 12;110(6 Pt 2):1301–3. 12456948

[5] Blum RW, Garell D, Hodgman CH, Jorissen TW, Okinow NA, Orr DP, et al Transition from child-centered to adult health-care systems for adolescents with chronic conditions. A position paper of the Society for Adolescent Medicine. J Adolesc Health Off Publ Soc Adolesc Med. 1993 11;14(7):570–6. 10.1016/1054-139x(93)90143-d 8312295

[6] Monaghan M, Hilliard M, Sweenie R, Riekert K. Transition Readiness in Adolescents and Emerging Adults with Diabetes: The Role of Patient-Provider Communication. Curr Diab Rep. 2013 12;13(6):900–8. 10.1007/s11892-013-0420-x 24014075 PMC3832624

[7] Bennett DL, Towns SJ, Steinbeck KS. Smoothing the transition to adult care. Med J Aust. 2005 4 18;182(8):373–4. 15850429 10.5694/j.1326-5377.2005.tb06751.x

[8] Watson AR. Problems and pitfalls of transition from paediatric to adult renal care. Pediatr Nephrol Berl Ger. 2005 2;20(2):113–7. 10.1007/s00467-004-1763-y 15627164

[9] Cadario F, Prodam F, Bellone S, Trada M, Binotti M, Trada M, et al Transition process of patients with type 1 diabetes (T1DM) from paediatric to the adult health care service: a hospital-based approach. Clin Endocrinol (Oxf). 2009 9;71(3):346–50. 19178523 10.1111/j.1365-2265.2008.03467.x

[10] Hersh AO, Pang S, Curran ML, Milojevic DS, von Scheven E. The challenges of transferring chronic illness patients to adult care: reflections from pediatric and adult rheumatology at a US academic center. Pediatr Rheumatol Online J. 2009;7:13 10.1186/1546-0096-7-13 19505332 PMC2700108

[11] Vaudre G, Sylvain H, Delmas P, Dollfus C, Leverger G. [Consequences and experiences of the transition to adult medicine for young people living with human immunodeficiency virus (HIV)]. Arch Pédiatrie Organe Off Sociéte Fr Pédiatrie. 2012 8;19(8):786–93. 10.1016/j.arcped.2012.05.003 22743171

[12] Fish R, Judd A, Jungmann E, O’Leary C, Foster C, HIV Young Persons Network (HYPNet). Mortality in perinatally HIV-infected young people in England following transition to adult care: an HIV Young Persons Network (HYPNet) audit. HIV Med. 2014 4;15(4):239–44. 10.1111/hiv.12091 24112550

[13] UNAIDS. Global Report UNAIDS—Report on the global AIDS epidemic 2013 [Internet]. 2013. Available from: http://www.unaids.org/sites/default/files/media_asset/UNAIDS_Global_Report_2013_en_1.pdf

[14] WHO. HIV and adolescents: guidance for HIV testing and counselling and care for adolescents living with HIV: recommendations for a public health approach and considerations for policy-makers and managers. 2013. 25032477

[15] Agwu AL, Fairlie L. Antiretroviral treatment, management challenges and outcomes in perinatally HIV-infected adolescents. J Int AIDS Soc. 2013;16:18579 10.7448/IAS.16.1.18579 23782477 PMC3687074

[16] Hussen SA, Chahroudi A, Boylan A, Camacho-Gonzalez AF, Hackett S, Chakraborty R. Transition of youth living with HIV from pediatric to adult-oriented healthcare: a review of the literature. Future Virol. 2015;9(10):921–9. 10.2217/fvl.14.73 25983853 PMC4433446

[17] Gilliam PP, Ellen JM, Leonard L, Kinsman S, Jevitt CM, Straub DM. Transition of Adolescents With HIV to Adult Care: Characteristics and Current Practices of the Adolescent Trials Network for HIV/AIDS Interventions. J Assoc Nurses AIDS Care. 2011 7;22(4):283–94. 10.1016/j.jana.2010.04.003 20541443 PMC3315706

[18] WHO. ADOLESCENT HIV TESTING, COUNSELLING AND CARE Implementation guidance for health providers and planners [Internet]. 2014. Available from: http://apps.who.int/adolescent/hiv-testing-treatment/page/Transition

[19] Transitioning HIV-Infected Adolescents Into Adult Care [Internet]. 2011. Available from: http://www.hivguidelines.org/clinical-guidelines/adolescents/transitioning-hiv-infected-adolescents-into-adult-care/

[20] Velter A, Barin F, Bouyssou A, Guinard J, Léon L, Le Vu S, et al HIV prevalence and sexual risk behaviors associated with awareness of HIV status among men who have sex with men in Paris, France. AIDS Behav. 2013 5;17(4):1266–78. 10.1007/s10461-012-0303-1 22968398

[21] INVS. http://www.invs.sante.fr/Dossiers-thematiques/Maladies-infectieuses/VIH-sida-IST/Infection-a-VIH-et-sida/Incidence-de-l-infection-par-le-VIH. 2013.

[22] Glaser BG, Strauss AL. The discovery of grounded theory: strategies for qualitative research. 4 paperback printing. New Brunswick: Aldine; 2009. 271 p.

[23] Paillé P, Mucchielli A. L’Analyse qualitative en sciences humaines et sociales. Armand Colin; 2008.

[24] Tong A, Sainsbury P, Craig J. Consolidated criteria for reporting qualitative research (COREQ): a 32-item checklist for interviews and focus groups. Int J Qual Health Care J Int Soc Qual Health Care ISQua. 2007 Dec;19(6):349–57. 10.1093/intqhc/mzm042 17872937

[25] Sohn AH, Hazra R. The changing epidemiology of the global paediatric HIV epidemic: keeping track of perinatally HIV-infected adolescents. J Int AIDS Soc [Internet]. 2013 6 18 [cited 2016 Oct 31];16(1). Available from: http://www.jiasociety.org/index.php/jias/article/view/18555 10.7448/IAS.16.1.18555 PMC3687075 23782474

[26] Hill TE. How clinicians make (or avoid) moral judgments of patients: implications of the evidence for relationships and research. Philos Ethics Humanit Med. 2010;5(1):11. 20618947 10.1186/1747-5341-5-11 PMC2914676

[27] Wasan AD, Wootton J, Jamison RN. Dealing with difficult patients in your pain practice. Reg Anesth Pain Med. 2005 4;30(2):184–92. 15765460 10.1016/j.rapm.2004.11.005

[28] Kelly MP, May D. Good and bad patients: a review of the literature and a theoretical critique. J Adv Nurs. 1982 3;7(2):147–56. 7045186 10.1111/j.1365-2648.1982.tb00222.x

[29] Orban LA, Stein R, Koenig LJ, Conner LC, Rexhouse EL, Lewis JV, et al Coping strategies of adolescents living with HIV: disease-specific stressors and responses. AIDS Care. 2010 4;22(4):420–30. 10.1080/09540120903193724 20146110

[30] Krebs EE, Garrett JM, Konrad TR. The difficult doctor? Characteristics of physicians who report frustration with patients: an analysis of survey data. BMC Health Serv Res. 2006;6:128 10.1186/1472-6963-6-128 17026762 PMC1617099

[31] Ingersoll KS, Heckman CJ. Patient-clinician relationships and treatment system effects on HIV medication adherence. AIDS Behav. 2005 3;9(1):89–101. 10.1007/s10461-005-1684-1 15812616 PMC2868061

[32] Newman C, Persson A, Miller A, Cama E. Bridging worlds, breaking rules: Clinician perspectives on transitioning young people with perinatally acquired HIV into adult care in a low prevalence setting. AIDS Patient Care STDs. 2014 7;28(7):381–93. 10.1089/apc.2013.0346 24749770

[33] Agwu AL, Lee L, Fleishman JA, Voss C, Yehia BR, Althoff KN, et al Aging and Loss to Follow-up Among Youth Living With Human Immunodeficiency Virus in the HIV Research Network. J Adolesc Health. 2015 3;56(3):345–51. 10.1016/j.jadohealth.2014.11.009 25703322 PMC4378241

[34] Arora S, Ashrafian H, Davis R, Athanasiou T, Darzi A, Sevdalis N. Emotional intelligence in medicine: a systematic review through the context of the ACGME competencies. Med Educ. 2010 8;44(8):749–64. 10.1111/j.1365-2923.2010.03709.x 20633215

[35] Mayer J. D., & Salovey P. (1997). What is emotional intelligence? In Salovey P. & Sluyter D. J. (Eds.), Emotional development and emotional intelligence: Educational implications (pp. 3–34). New York: Harper Collins. In.

[36] Bodenlos JS, Grothe KB, Whitehead D, Konkle-Parker DJ, Jones GN, Brantley PJ. Attitudes toward health care providers and appointment attendance in HIV/AIDS patients. J Assoc Nurses AIDS Care JANAC. 2007 6;18(3):65–73. 10.1016/j.jana.2007.03.002 17570301

