# Supplementary material for: Professional’s Perspectives on Care Management of Young People with Perinatally Acquired HIV during Transition: A Qualitative Study in Adult Care Setting
Source: PLoS One. 2017 Jan 23;12(1):e0169782. doi: 10.1371/journal.pone.0169782 (PMC5256933; doi:10.1371/journal.pone.0169782)
Supplement: S1 Checklist — (DOCX) [file pone.0169782.s001.docx]

**S1 Checklist. Consolidated criteria for reporting qualitative studies (COREQ) – Checklist.**

| **Domain 1: Research team and reflexivity** | |
| --- | --- |
| ***Personal characteristics*** | |
| 1. Which author(s) conducted the interview? | Interviews were conducted by SG and EL with 11 participants and EL only with 7 participants |
| 1. What were the researcher’s credentials? | EL: PhD candidate in public health; SG: MD |
| 1. What was their occupation at the time of the study? | EL: PhD candidate; SG: researcher in public health |
| 1. Was the researcher male or female? | EL : female, SG : male |
| 1. What experience or training did the researcher have | EL: master degree formation in conducting qualitative research SG: professional experience in conducting qualitative research |
| ***Relationship with participants*** | |
| 1. Was a relationship established prior to study commencement | EL did not know the participants before the study. SG had worked on previous projects (unrelated to the subject of study) with two interviewed medical doctors |
| 1. What did the participants know about the researcher? | The researchers presented the study and the hospital and the research team to which they belonged |
| 1. What characteristics were reported about the interviewer/facilitator? | The occupation of researcher and their implication in the study were reported to participants |
| **Domain 2: Study design** | |
| ***Theoretical framework / Participant selection*** | |
| 1. What methodological orientation was stated to underpin the study? | Thematic analysis was used based on inductive approach |
| 1. How were the participants selected? | Participants were recruited for their experience in transition of care with HIV young through snowball recruitment. A diversity of profiles was researched. |
| 1. How were the participants approached? | Participants were approached by e-mails |
| 1. How many participants were in the study? | There were 18 participants |
| 1. How many participants refused to participate or dropped out? Why? | 2 : 1 nurse did not responded, 1 patient association responded that their population do not match with our studied problematic |
| ***Setting*** | |
| 1. Where was the data collected? | Interviews were conducted on participant’s workplaces |
| 1. Was anyone else present besides the participants and researcher? | Nobody else was present during interviews |
| 1. What are the important characteristics of the sample? | Participants were from various structures: Parisian and suburban hospitals, teaching or not, hospitals in which there were a paediatric service in addition to adult services or not, an health unit for young adults and a patient association. All categories of professionals involved in the transition have been invited. |
| ***Data collection*** | |
| 1. Were questions, prompts, guides provided by the author? Was it pilot tested? | The interview guide was developed by reviewing the transition-related literature, and was adapted after first interviews with professionals |
| 1. Were repeat interviews carried out? | No repeat interviews were carried out |
| 1. Did the research use audio or visual recording to collect the data? | 11/16 interviews were recorded. The remainder of the interviews were not recorded (due to equipment-related problems or preferences of participants) but were conducted by two researchers who took detailed notes. |
| 1. Were field notes made during and/or after the interview or focus group? | Notes were taken during each interview. After each interview conducted by two researchers, notes were compared and compiled |
| 1. What was the duration of interviews or focus groups? | The interviews ranged in duration from 25 to 70 minutes. |
| 1. Was data saturation discussed? | Data saturation was discussed between EL and SG. The choice to stop the recruitment was consensual. |
| 1. Were transcripts returned to participants for comments and/or correction? | Transcripts were not returned to participants |
| **Domain 3: Analysis and findings** | |
| ***Data analysis*** | |
| 1. How many data coders coded the data? | Transcripts were coded independently by EL and SG. Emerging themes and analytical framework were discussed to reach a consensus |
| 1. Did authors provide a description of the coding tree? | We do not present the coding tree |
| 1. Were themes identified in advance or derived from the data? | The themes were derived from the data |
| 1. What software, if applicable, was used to manage the data? | The analysis of the verbatim transcripts was structured using Nvivo10 software |
| 1. Did participants provide feedback on the findings? | No feedback was obtained from participants but feedback was obtained from members of a workshop on the theme “HIV-positive teenagers |
| ***Reporting*** | |
| 1. Were participant quotations presented to illustrate the themes/findings? Was each quotation identified? | We present some quotations to illustrate findings. The profession and the workplace of the interviewee were mentioned below. |
| 1. Was there consistency between the data presented and the findings | The data presented and the findings are consistent |
| 1. Were major themes clearly presented in the findings? | Paragraph structure of “results” part was built to address each major theme |
| 1. Is there a description of diverse cases or discussion of minor themes? | We report and describe diverse cases |
